# Changes in neonatal morbidity, neonatal care practices, and length of hospital stay of surviving infants born very preterm in the Netherlands in the 1980s and in the 2000s: a comparison analysis with identical characteristics definitions

**DOI:** 10.1186/s12887-023-04354-x

**Published:** 2023-11-04

**Authors:** Réka E. Sexty, Sylvia van der Pal, Sijmen A. Reijneveld, Dieter Wolke, Guido Lüchters, Leonhard Bakker, Stef van Buuren, Arend F. Bos, Peter Bartmann

**Affiliations:** 1 Department of Psychology, Health Psychology Unit, University of Graz, Graz, Austria; 2 University Hospital Bonn, Children’s Hospital, Bonn, Germany; 3 TNO, Child Health, Leiden, the Netherlands; 4 Department of Health Sciences, University Medical Center Groningen, University of Groningen, Groningen, the Netherlands; 5 Department of Psychology, University of Warwick, Coventry, UK; 6 Centre for Development Research (ZEF), Biostatistics, Bonn, Germany; 7 Department of Paediatrics, University Medical Center Groningen, University of Groningen, Groningen, the Netherlands

**Keywords:** Very preterm infant, Neonatal morbidity, Neonatal care practices, POPS, LOLLIPOP, RECAP preterm

## Abstract

**Background:**

This study evaluates changes in the neonatal morbidity, the neonatal care practices, and the length of hospital stay of surviving very preterm (VP) infants born in the Netherlands in the 1980s and in the 2000s; a period over which historical improvements were introduced into neonatal care. We, herein, also study whether these changes in neonatal morbidity, neonatal care practices and length of hospital stay are associated with sociodemographic, prenatal, and infant characteristics.

**Methods:**

Two community-based cohorts from 1983 (POPS) and 2002−03 (LOLLIPOP) have provided the perinatal data for our study. The analysis enrolled 1,228 participants born VP (before the 32nd week of gestation) and surviving to 2 years of age without any severe congenital malformation. A rigorous harmonisation protocol ensured a precise comparison of the cohorts by using identical definitions of the perinatal characteristics.

**Results:**

In 2003, mothers were older when giving birth, had higher multiple birth rates, and significantly more parents had received higher education. In 2003, less VP infants had severe intraventricular haemorrhage and sepsis and relatively more received continuous positive airway pressure, mechanical ventilation and caffeine therapy than in 1983. Antenatal corticosteroids and surfactant therapy were provided only in 2003. The length of the stay in the neonatal intensive care unit and in hospital had decreased in 2003 by 22 and 11 days, respectively. Differences persisted after adjustment for sociodemographic, prenatal, and infant characteristics.

**Conclusions:**

Neonatal morbidities of the surviving VP infants in this study have not increased, and exhibit improvements for various characteristics in two cohorts born 20 years apart with comparable gestational age and birth weight. Our data suggest that the improvements found are associated with more advanced therapeutic approaches and new national protocols in place, and less so with sociodemographic changes. This analysis provides a basis for further comparative analyses of the health and the development of VP children, particularly with regard to long-term outcomes.

**Supplementary Information:**

The online version contains supplementary material available at 10.1186/s12887-023-04354-x.

## Background

Very preterm (VP) birth (i.e., before the 32 weeks of gestation) is a significant public health concern globally, and it is associated with high rates of mortality as well as short- and long-term morbidities [1]. VP birth can result in neurodevelopmental, behavioural, and organ-specific health problems persisting throughout childhood and into adulthood [2]. The sequelae of preterm birth can put a high burden on the family of the child, the health care system, and society [1]. There is consistent evidence that mortality decreased over the decades into the 2000s, mainly in the group of extremely low birth weight / extremely preterm infants, and at gestational ages at the limit of viability [3–7]. In the Netherlands, mortality of VP infants decreased from 25.4% to 1983 to 20.0% in 1995 [8] and 18.1% in 2002 [9]. The urgent question is whether this decrease in mortality might have led to a higher morbidity in the surviving infants.

Most studies conducted throughout different eras report an increase in infants surviving without major neonatal morbidities [3–6]. Mixed outcome results were observed for single major morbidities like bronchopulmonary dysplasia (BPD), severe intraventricular haemorrhage (IVH), early and late-onset sepsis, severe retinopathy of prematurity (ROP), necrotising enterocolitis (NEC) or patent ductus arteriosus [3–7, 10, 11].

Introduction of new obstetric and neonatal/paediatric care practices in the 1990s [3–5] that are nowadays considered as the most effective evidence-based practices [12] might also explain improvements in neonatal morbidities of survivors. Antenatal corticosteroids given to women at risk of a VP delivery were implemented in order to promote foetal lung maturation. The use of antenatal corticosteroids has not only reduced mortality rates, but has also resulted in fewer neonates suffering from respiratory distress syndrome and IVH [4]. Meanwhile, postnatal care introduced the use of nasal continuous positive airway pressure (CPAP), new ventilation techniques and most importantly, the intratracheal administration of surfactant [4, 11]. In the 1990s, there was a trend in most national health care systems to establish and centralise neonatal intensive care units (NICUs), thereby providing professional and specialised care for VP-born infants [4].

However, not only obstetric and neonatal care has changed, but there may also be changes in social conditions, such as higher educated mothers living in improved social circumstances and leading a healthier lifestyle (including fewer mothers smoking during pregnancy). Social factors have been found to be associated with both prenatal and neonatal morbidities as well as with infant outcome [13, 14].

Evidence that neonatal morbidity still varies considerably across different regions in Europe [15, 16] underlines the need to compare data at a national level, from cohorts recruited more than a decade apart. For this purpose, we have conducted a comparison of two community-based cohorts within only one country, namely the Netherlands: Project on Preterm and Small-for-gestational age infants (POPS, 1983) and Longitudinal Preterm Outcome Project (LOLLIPOP, 2002−03). Between 1983 and 2002−03, three new policies and acts have been passed and implemented in neonatal care in the Netherlands. First, new modalities such as antenatal corticosteroids, surfactant therapy, and high frequency ventilation were introduced [17]. Second, the Act of the Dutch Ministry of Health [18] has assigned 10 centres for neonatal intensive care treatment, while the Health Council [19] has recommended obstetrical staff to transfer pregnant women with risk of premature birth to perinatal centres (centralisation). Third, the care of extremely preterm infants was conservative before the 2000s [20] [21] in terms that the obstetrical guidelines focused on the prolongation of pregnancy and not an increase of the number of live births. Intensive neonatal treatment for infants born < 26 weeks is only recommended in the Netherlands since 2005 [21]. Because of considerable changes in neonatal care practices and national policies associated with improved survival, but still unclear improvement in neonatal morbidities of VP infants, there is a need for investigations to assess changes in neonatal outcomes and treatments throughout different eras. POPS and LOLLIPOP with community-based data from the same national background provide this opportunity. Therefore, this study aimed at identifying: (i) changes in neonatal morbidity, neonatal care practices, and the length of NICU and hospital stay of infants born VP between 1983 and 2003 in the Netherlands, and (ii) whether these changes are associated with sociodemographic and prenatal characteristics of the mother as well as with neonatal characteristics of the infant.

## Methods

### Study design

This is an observational study using data collected during the neonatal period in two Dutch cohorts of VP infants from 1983 (POPS) and 2002−03 (LOLLIPOP). This study followed the Strengthening the Reporting of Observational studies in Epidemiology (STROBE) guidelines for reporting cohort studies [22]. The cohort data were made available by using data transfer agreements between the partners.

### Study populations

#### POPS cohort (1983)

The POPS cohort prospectively comprised of 1,336 VP and/or very low birth weight (VLBW; <1,500-g) infants who were born alive in the Netherlands between January 1 and December 31, 1983. The study population consisted of 94% of all infants born VP/VLBW in the Netherlands [23]. The paediatricians of the neonatal units of the participating hospitals completed an extensive, standardised, pre-coded list of perinatal data until discharge [17]. The data collection of the cohort is still ongoing, and the participants took part at the last wave of data collection in 2018−19 [24].

#### LOLLIPOP cohort (2002−03)

The LOLLIPOP is a community-based cohort originally including preterm and full-term children born in 2002 and in 2003 [25, 26]. Thirteen preventive child healthcare centres (PCHCs) participated in the study. All children attending the last PCHC visit at age 43−49 months and born before 37 weeks of gestation were included (approximately 25% of this age group population in the Netherlands). The VP children born in 2003, hospitalised in one of the five participating NICUs, and who were alive at the follow-up were also included in the study group. With regards to the national birth cohorts of the Netherlands, the initial sample of the LOLLIPOP cohort was fairly representative of the entire population. Children with major congenital malformations were excluded. Perinatal and neonatal data were collected from registered data from various sources, including a general parental questionnaire, birth registers, PCHC records and medical records of both the mother and the child, thereby allowing for a cross-checking of the collected information.

#### Ethics of the studies

Medical Ethic Committees of the participating hospitals have approved the study protocol for POPS [23], while the Medical Ethic Committee of the University Medical Center Groningen has approved the study protocol for LOLLIPOP [26]. All participating parents have provided written informed consent.

### Harmonised data

Perinatal data was harmonised retrospectively, following the RECAP preterm harmonisation guidelines [27] as well as other sources reporting harmonisation techniques [28, 29]. First, a set of target variables relevant to the research questions was defined (Supplementary File 1). Second, definitions, value units, and variable categories were checked regarding the adequacy and the possible concordance or overlap with the definition of the variables of the other cohort. Third, variables were organised into three categories depending on their eligibility for harmonisation. Due to incompatible definitions of various characteristics applied in the two studies, some items of interest (such as BPD) could not be evaluated. After generating a joint harmonised dataset, descriptive statistics were calculated and variables with large percentages of missing data (> 40%) were excluded from our analyses. Table 1 presents the variables harmonised for the comparison analysis.

**Table 1:** Variables harmonised for the comparison analysis

Maternal and infant characteristics potentially affecting outcomes		Outcomes		
**Maternal characteristics**	**Infant characteristics**	**Neonatal morbidity**	**Neonatal care practices**	
**Sociodemographic characteristics**: • age at birth • parental education **Prenatal characteristics**: • previous birth • smoking during pregnancy • antenatal corticosteroids	• GA • BW • SGA • sex • multiples • PPROM • meconium-stained amniotic fluid • breech presentation • APGAR at 5 min < 7 • cesarean section	• IVH grade III-IV • proven NEC • sepsis • apnea	• mechanical ventilation • CPAP • caffeine therapy • postnatal corticosteroids • length of mechanical ventilation • surfactant therapy	• length of stay in NICU • length of stay in hospital

GA: gestational age, BW: birth weight, SGA: small for gestation, PPROM: preterm premature rupture of membranes, IVH: intraventricular hemorrhage, NEC: necrotizing enterocolitis, CPAP: continuous positive airway pressure, NICU: neonatal intensive care unit

### Statistical analysis

Cohorts were compared in terms of sociodemographic and prenatal characteristics, as well as in terms of infant characteristics, by applying independent *t*-tests for continuous variables and chi-square tests for categorical variables.

In order to test for differences between cohorts on neonatal morbidity and neonatal care practices, multiple negative binomial regression models were applied for continuous variables without normal distribution [30], and multiple logistic regression models were applied for dichotomous variables. Adjusted models included covariates, such as sociodemographic, prenatal and infant characteristics. Additionally, differences on length of the NICU and the hospital stay were adjusted for all neonatal morbidity and the care practices used in the study.

Analyses were based on participants with non-missing values on covariates and outcomes. A *p*-value of < 0.05 was considered as statistically significant; in case of multiple testing the *p*-value was Bonferroni-corrected. The statistical software packages SPSS 27.0 (IBM SPSS for Windows) and Stata 16.1 (Statacorp. Stata Statistical Software) were used for the undertaking of the calculations.

## Results

### Participants: harmonisation of the two cohorts

**Figure 1:**
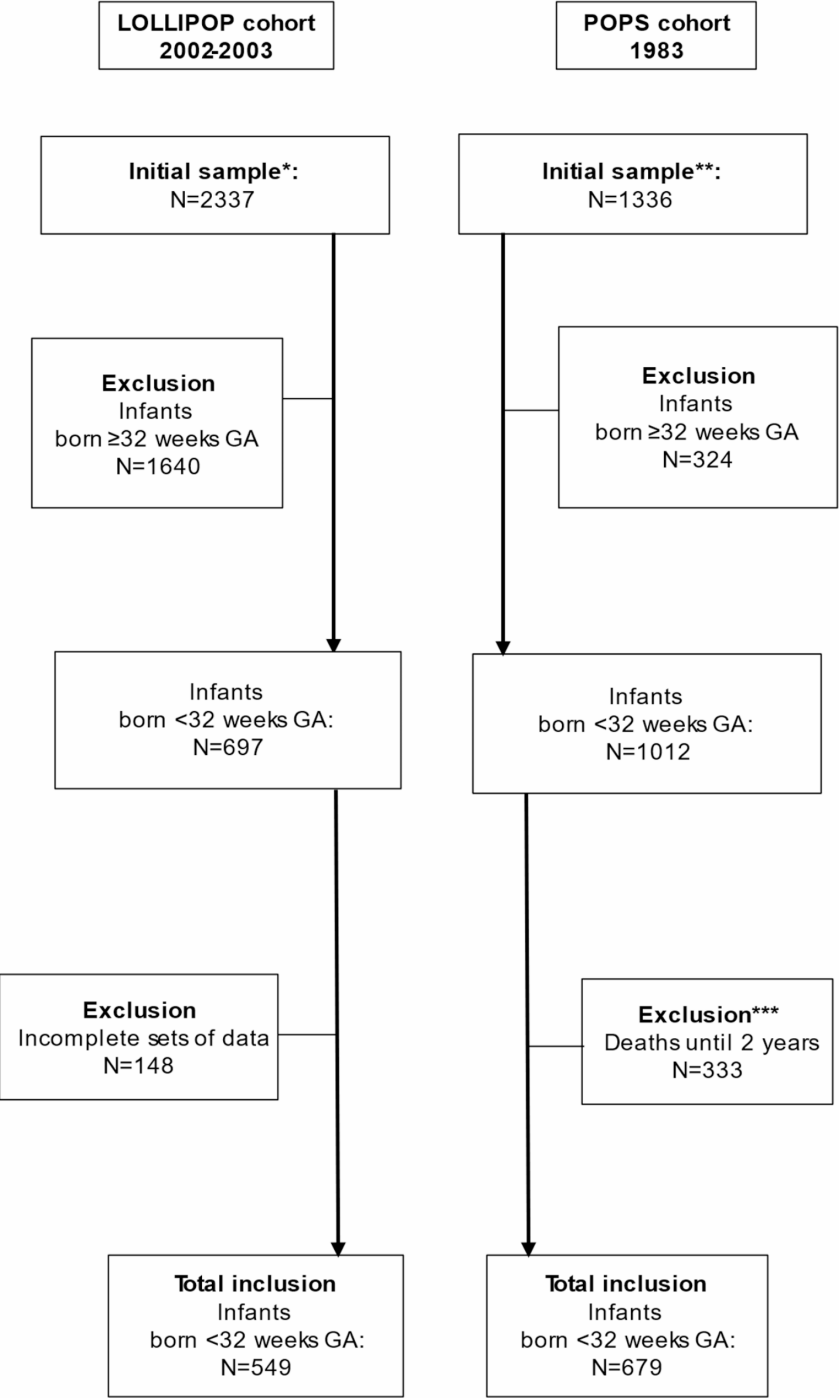
Flowchart: participant inclusion. * Initial LOLLIPOP sample comprised 4 year-old surviving preterm children without major congenital malformations. ** Initial POPS sample comprised 94% of VP infants born in 1983 in the Netherlands. *** All cases with severe congenital malformations died before 2 years of age

As POPS included infants born VP (< 32 weeks of gestational age or GA) or VLBW (< 1,500 g) and LOLLIPOP included only infants born at < 37 weeks GA, we applied the following joint inclusion criteria for the present analysis: (i) a GA at birth being < 32 weeks, (ii) the absence of any severe congenital malformations, (iii) a confirmed survival of up to 2 years, and (iv) the availability of complete perinatal data. Figure 1 provides an overview of how participants of both cohorts were selected. The final dataset comprised N = 679 infants from POPS (67% of the POPS infants born < 32 weeks GA) and N = 549 infants from LOLLIPOP (79% of the LOLLIPOP infants born < 32 weeks GA).

### Description of the cohorts

#### Maternal and infant characteristics

Table 2 summarises for the two cohorts the sociodemographic, prenatal, and infant characteristics. LOLLIPOP mothers were more highly educated and older at birth (mean: 3.3 years) than the POPS ones. Fewer mothers from LOLLIPOP reported smoking during pregnancy, and they had fewer previous births than the mothers of the POPS study.

**Table 2:** Comparison analysis between cohorts on maternal and infant characteristics

	LOLLIPOP (2002-03) N = 549		POPS (1983) N = 679			
Maternal characteristics - Sociodemographic characteristics						
	Mean	SD	Mean	SD	T-value (df)	P-value
Maternal age at birth, years	30.5	4.5	27.2	4.7	9.18 (363)	< 0.0001*
	**N**	**%**	**N**	**%**	**Chi** ^**2**^ **(df)**	
Parental education, high	227	41.9	158	25.4	64.8 (2)	< 0.0001*
**Maternal characteristics - Prenatal characteristics**						
Previous birth	144	26.2	383	56.5	113.4 (1)	< 0.0001*
Maternal smoking during pregnancy	112	20.5	190	31.4	17.5 (1)	< 0.0001*
**Infant characteristics**						
	**Mean**	**SD**	**Mean**	**SD**	**T-value (df)**	
Gestational age, weeks	29.2	1.6	29.4	1.5	1.8 (1226)	0.07
Birth weight, grams	1298.2	367.4	1329.2	315.5	1.6 (1226)	0.11
	** N**	**%**	**N**	**%**	**Chi** ^**2**^ **(df)**	
SGA	104	18.9	110	16.2	1.6 (1)	0.21
Sex, male	265	48.3	363	53.9	0.6 (1)	0.55
Multiples	187	34.1	156	23.0	18.5 (1)	< 0.0001*
PPROM	95	17.9	163	24.0	6.6 (1)	0.010
Meconium stained amniotic fluid	12	2.3	33	5.0	6.1 (1)	0.014
Breech presentation	124	36.3	187	27.5	9.4 (1)	0.002
APGAR < 7 at 5 min	46	8.7	80	13.4	6.5 (1)	0.011
Born with cesarean section	282	53.1	242	35.6	37.0 (1)	< 0.0001*

* still significant after Bonferroni correction

In POPS, complete data ranged between N = 595 and N = 679 depending on characteristic. In LOLLIPOP, complete data ranged between N = 531 and N = 549, except for maternal age (N = 211) and breech presentation (N = 424)

There were no differences in terms of the infant GA, birth weight (BW), small-for-gestational age (SGA) status, and sex between the cohorts. Preterm premature rupture of membranes (PPROM), meconium-stained amniotic fluid, and low AGPAR score at 5 min were less frequent in LOLLIPOP participants when compared to those of POPS. LOLLIPOP infants were also more likely to be born as multiples and *via* a caesarean section.

### Main outcomes

#### Neonatal morbidity, neonatal care practices and length of hospital stay

Table 3, model 1, shows the unadjusted differences in neonatal morbidity, neonatal care practices and the length of hospital stay between the two cohorts. The LOLLIPOP infants were less frequently diagnosed with severe IVH and sepsis, but more frequently exhibited apnoeic events than POPS infants. There was no significant difference in the prevalence of NEC. We also found that LOLLIPOP infants were more likely to receive mechanical ventilation, CPAP and caffeine therapy. Although more infants were treated with mechanical ventilation in LOLLIPOP, the length of the ventilation remained unchanged between the two periods examined. The length of stay in the NICU was 57% shorter in LOLLIPOP (median: 16 days) than in POPS (median: 38 days). LOLLIPOP infants also spent 11 days less (in terms of total time) in the hospital after birth. This is a reduction of 17% when compared to that of POPS.

**Table 3:** Differences in neonatal morbidity and care practices of VP-born infants from the LOLLIPOP and POPS cohorts

	LOLLIPOP (2002-03) N = 549		POPS (1983) N = 679		Effect of cohort (LOLLIPOP vs. POPS)			
					Model 1	Model 2	Model 3	Model 4
	N	%	N	%	OR (CI 95%)	OR (CI 95%)	OR (CI 95%)	OR (CI 95%)
**Morbidity**								
IVH grade III-IV	18	3.6	47	9.3	0.36 (0.21–0.33)***	0.15 (0.04–0.50)**	0.15 (0.04–0.55)**	0.26 (0.06–1.12)(*)
sepsis	135	27.7	246	36.3	0.67 (0.52–0.87)**	0.59 (0-40-0.86)**	0.63 (0.42–0.96)*	0.53 (0.32–0.88)*
proven NEC	12	2.3	11	1.6	1.42 (0.62–3.23)	1.37 (0.38–5.04)	1.56 (0.36–6.76)	2.22 (0.45–11.10)
apnea	465	90.8	453	66.9	4.89 (3.48–6.87)***	6.02 (3.47–10.46)***	6.50 (3.65–11.57)***	10.52 (5.18–21.34)***
**Care practices**								
Surfactant therapy	191	34.8						
mechanical ventilation	291	55.7	302	47.0	1.42 (1.13-1-79)**	1.22 (0.88–1.72)	1.40 (0.97–2.03)(*)	1.52 (0.96–2.42)(*)
CPAP	438	83.9	305	46.9	5.90 (4.46–7.80)***	6.99 (4.50-10.86)***	8.36 (5.22–13.37)***	7.88 (4.64–13.37)***
caffeine therapy	452	89.3	402	64.1	4.68 (3.38–6.49)***	5.07 (3.07–8.36)***	5.62 (3.33–9.48)***	7.93 (4.26–14.77)***
postnatal corticosteroids	28	5.5	49	7.2	0.76 (0.45–1.20)	0.46 (0.20–1.07)(*)	0.49 (0.20–1.18)	0.54 (0.18–1.57)
	**Median**	**Range**	**Median**	**Range**	**Model 1** **IRR (CI 95%)**	**Model 2** **IRR (CI 95%)**	**Model 3** **IRR (CI 95%)**	**Model 4** **IRR (CI 95%)**
length of mechanical ventilation, days	1	0–84	0	0–78	1.15 (0.91–1.46)	1.10 (0.76–1.61)	1.16 (0.76–1.78)	0.92 (0.57–1.46)
length of NICU stay, days	16	0-143	38	0-380	0.48 (0.44–0.54)***	0.44 (0.38–0.51)***	0.41 (0.35–0.48)***	0.39 (0.33–0.46)***
length of hospital stay, days	54	10–339	65	12–380	0.86 (0.82–0.90)***	0.86 (0.81–0.92)***	0.83 (0.78–0.90)***	0.89 (0.83–0.94)***

OR: odds ratio IRR: incidence rate ratio *** p < 0.001 **p < 0.01 *p < 0.05 (*)p < 0.1

Model 1: unadjusted

Model 2: adjusted for sociodemographic characteristics (maternal age at birth, parental education)

Model 3 Model 2 + adjusted for prenatal characteristics (maternal smoking during pregnancy, previous pregnancy)

Model 4: Model 3 + adjusted for infant characteristics (gestational age, small for gestation, multiple birth, sex, meconium, PPROM, breech presentation, low APGAR, cesarean section)

In POPS, complete data ranged from N = 627 to N = 679, but IVH comprised N = 505 complete data, and length of NICU stay N = 454. In LOLLIPOP, complete data ranged from N = 462 to N = 526

Second, we assessed the differences in neonatal morbidity, neonatal care practices and the length of hospital stay between the two cohorts, when outcomes are adjusted for sociodemographic (Model 2) and prenatal characteristics (Model 3), as well as for infant characteristics (Model 4). The odds ratios (OR) for apnoea and sepsis remained significant, but the confidence intervals (CI) increased in the adjusted models. After entering the infant characteristics into the regression model, the low GA and the low APGAR scores displayed a strong association with the occurrence of sepsis and apnoea (data not shown). The significant difference between the rates of severe IVH in the two cohorts became a trend (not significant) when the infant characteristics were considered. The low GA exhibited an unequivocal connection to severe IVH (data not shown). The differences in neonatal care practices over time remained and increased for the rate of CPAP and caffeine therapy, after adjustment for infant and maternal characteristics. Additionally, the low GA was associated with use of both care practices (data not shown).

The lengths of NICU and hospital stay were also analysed with adjustment for all neonatal morbidity and care practices; OR: 0.38, 95% CI: 0.32−0.46, *p* = 0.000 and OR: 0.87, 95% CI: 0.81−0.94, *p* = 0.000, respectively. Adjustments did not change the significant differences with regard to the length of the NICU and the hospital stay. Additionally, low GA, SGA, caesarean section, mechanical ventilation, and postnatal corticosteroids exhibited an association with the increase of the length of the NICU and the hospital stay.

## Discussion

The most important finding of this study was a significant decrease in the duration of NICU and hospital stay of VP-born infants in the Netherlands between 1983 and 2003. Additionally, changes in rates of neonatal morbidities and care practices were also observed: declined IVH- and sepsis-rates, and increased rates of apnoea and a more common use of CPAP and caffeine therapy in the 2000s,

### Sociodemographic and prenatal characteristics

We observed a higher maternal age (30.5 vs. 27.2 years) and a higher parental education (41.9% vs. 25.4%) in LOLLIPOP, which can be explained by a general sociodemographic trend in Western Europe [31, 32]. In the Netherlands maternal age at first childbirth increased from 28 years of age in 1970 to 30 years in 2000 and the rate of tertiary education rose from 22,2% (1990) to 32.1% in 2003. The decline of maternal smoking during pregnancy from 31.4% (POPS) to 20.5% (LOLLIPOP) could be expected, as the prevalence of daily smoking of Dutch adults decreased from 40% to 1983 to 26.7% in 2003 [33]. Meanwhile, the general trend for total fertility rate increased from 1.47 to 1.75 between 1983 and 2003 in the Netherlands [34]. In this study, we observed more primipara in LOLLIPOP.

### Infant characteristics

In both cohorts, the prevalence of infants born before 26 weeks GA was under 2%. This phenomenon can be attributed to the fact that the policy on the treatment of VP infants was still conservative at the beginning of the 2000s in the Netherlands, with neonatal intensive care not routinely provided to infants born earlier than 26 weeks GA [20]. The number of multiple births was higher in the LOLLIPOP cohort. This is in line with a general trend of increased rates of multiple births associated with the increased use of assisted reproductive technologies (where multiple embryos were implanted) [4] and the increasing age of the mothers [35]. We observed improved obstetrical outcomes of infants born in the 2000s in terms of the rate of PPROM, meconium-stained amniotic fluid and low APGAR score when compared to the POPS infants. This may be attributed to the improved pregnancy care. The incidence of caesarean sections increased over time, and fits into the international trends [36].

### Neonatal morbidity

Our study revealed that the incidence of severe IVH and sepsis decreased in VP survivors over the two decades examined. The diagnosis of apnoea increased significantly from 66.9% (in 1983) to 90.8% (in 2003). Changes in the rates of some neonatal morbidities can be attributed to new routine care practices established between 1983 and 2003. The routine antenatal administration of corticosteroids to mothers with a risk of premature delivery may have played an important role in reducing the IVH rates [4]. Our analysis shows that the cohort difference disappeared after adjusting for infant characteristics. This result corresponds with the findings that infant characteristics such as the higher APGAR score, and the caesarean section can be associated with the decline in severe IVH rates [37]. Our study has revealed that low GA was a stronger risk factor for severe IVH than the cohort itself. Nevertheless, previous studies from the Netherlands have not reported any improved incidence of severe IVH in surviving VP infants between the 1980s and the 1990s (incidence rate of severe IVH was approximately 8% in the 1990s) [8, 38, 39].

We found an incidence of 27.7% of sepsis in 2003. This rate corresponds with results of other studies reporting late-onset sepsis incidence in the 2000s [10, 40], but it seems to be high when compared with a population-based finding from Switzerland [6]. Other authors reported a decrease in both early- and late-onset sepsis in the tertiary hospitals with NICU in the Netherlands between the 1980 and 2000 s which could be associated with group B Streptococcus prophylaxis by giving antibiotics to mothers with imminent preterm birth [41]. In addition, better availability of alcohol-based hand rubs should have become more common by the 2000s. Although, the lack of a general protocol on hand hygiene caused differences in various NICUs how sufficiently hospital workers used hand hygiene before contacting VP infants [42, 43].

The almost 100% occurrence of apnoea may be due to the increased use of CPAP instead of mechanical ventilation (see Neonatal care practices section),but could also be due to an increased awareness of apnoeic events and of the necessity of them being treated. The use of an accurate automated computer algorithm for detecting apnoea is a more reliable measure than the medical record that was mainly used in the previous years [44]. At the same time, the epidemiology of apnoea remains controversial [45]. Due to the different definitions for BPD used in the cohorts (diagnosis at postnatal 28 days *versus* 36 weeks postmenstrual age), this study could not analyse the collected BPD data. Only the duration of mechanical ventilation, as a possible factor affecting the BPD rates, was documented here, and it remained constant between 1983 and 2003. Anthony et al. [8]. have reported an increase in mean ventilatory days in the Netherlands: from 8.6 days in 1983 (POPS) to 14.2 days in 1995. However, this result can neither confirm nor reject the findings of other studies conducted in Europe that have reported an incidence of BPD around 10–20% [46] or exceeding 40% in the 2000s [47, 48].

### Neonatal care practices

There is good evidence that four neonatal care practices (namely birth in a tertiary centre with a NICU, the administration of antenatal corticosteroids, the prevention of hypothermia, and surfactant applied within 2 h after birth or early nasal CPAP) can result in survival with less severe morbidity for infants at high risk [12]. One of the four basic neonatal care practices (i.e., CPAP) was added to our analysis. We have found an increased use of this type of respiratory support in the LOLLIPOP cohort (increase of almost 40%). More LOLLIPOP infants received mechanical ventilation as well, but the increased rate of mechanical ventilation was less than 10%. CPAP was introduced into the neonatal care practices in the 1970s [49, 50], but it was still not used on a large scale in the 1980s when still mechanical ventilation was the primary treatment of respiratory failure of VPs [51]. Between the 1980 and 2000 s, several studies found that bubble CPAP at the delivery room could both prevent mechanical ventilation and reduce ventilatory induced lung damage [52, 53]. Additionally, the harmful effect of mechanical ventilation on neurodevelopment was meanwhile also reported [54]. This evidence should have resulted in a leading role of CPAP in the neonatal routine care by the 2000s.

We observed an increased rate of use of caffeine therapy in LOLLIPOP indeed. While in the 1980s, both theophylline and caffeine were used for treating apnoeic events, the largest trial about the beneficial effect of caffeine therapy in preventing BPD and neurodevelopmental impairments at 18 months of age was published in 2006 [55, 56]. As a RCT in 1992 reported that caffeine was more effective than theophylline in reducing apnoea [57], we can assume that caffeine therapy increasingly became an important part of neonatal care practices by the 2000s.

Therapies accelerating lung maturation and supporting lung function were administered only in the LOLLIPOP (and not in the POPS) at a proportion of 53.4% for complete antenatal corticosteroid treatment and 37.8% for receiving surfactant therapy. The rate of the use of a surfactant therapy is in line with [6] or below [5, 10] the average proportion of other findings from the 2000s. Other studies have reported both complete and incomplete steroid treatments received by the mother [10, 58] that can explain the lower proportion of full courses of antenatal corticosteroids administered in LOLLIPOP.

### Length of hospital stay

This study shows a significant decline in the length of NICU and of total hospital stay after a VP birth in 2003. The EuroHOPE Study [59] has compared the length of hospital stay of VP/VLBW infants from seven European countries between 2006 and 2008. Surviving infants spent between 46.2 and 61 days in the hospital until their first discharge. For the Netherlands, a median of 53.4 days is reported, which compares well with the 54 days observed here for the LOLLIPOP study (2003).

This difference in the length of NICU and hospital stay between the 1980s’ and the 2000s’ cohorts remained significant after an adjustment for neonatal morbidity and care practices. Thus, the study does not confirm that the length of the NICU and the hospital stay are closely connected to neonatal morbidity and care practices as analysed. The decline may be caused by the national protocols that were introduced between the two timepoints, e.g., new routines in the perinatal care and centralisation of neonatal institutions. In a European cross-country comparison, Maier et al. [60]. revealed that the median hospital stay after a VP birth was 51 days (range: 41−71 days) in 2003 in the Eastern-Central region of the Netherlands. This was below the average hospital stay in the European regions investigated (median: 56 days, range: 41–77 days). The authors have argued that this result might be explained by not providing an active management for infants born at before 26 weeks GA.

Our findings show that neonatal morbidities and care practices studied in this paper cannot explain the differences between cohorts in length of NICU and hospital stay. At the same time, we have to emphasise the decreasing rate of severe IVH and sepsis over time. In this study, we were not able to analyse potential changes in the incidence of various neonatal morbidities. There is evidence that BPD, ROP and periventricular leukomalacia became less frequent in the 2000s [40]. The more frequent use of CPAP and caffeine therapy could indicate a faster and more effective treatment of respiratory problems which could have resulted in fewer days spent in the hospital after birth. In the frame of this study, we cannot explain possible effects of other neonatal care practices such as feeding with human milk, community nursing, more discharges on partial tube feeds or the modification of discharge criteria. Additionally, specific maternal characteristics such as SES and maternal illness could have influenced preterm birth [61] and thus the development of neonatal morbidities and the length of hospital stay.

### Strengths, limitations, and implication for further research

As a strength, this study has compared data collected in the same country, with an identical population background, at two timepoints with a considerable difference of 20 years. The two cohorts represent a sizeable proportion of VP infants in the Netherlands in the respective years (POPS: 94%; LOLLIPOP: 25%). The study has also analysed data of VP infants who survived at least until 2 years of age. Finally, a rigorous protocol for the harmonisation of all variables selected for this study was followed. As a result of the harmonisation employed herein, only characteristics with identical definitions were considered eligible for analysis. The harmonisation we carried out forms a basis for undertaking further comparative analyses between POPS and LOLLIPOP that focus on the developmental outcomes of VP-born children in the future.

Among the limitations of our study are the differences of the two cohorts in terms of their research goals and the data collection methods followed. LOLLIPOP is a cohort with inclusion at age of 4 with retrospective data collection of perinatal and other follow-up data. This has resulted in missing data on the early life period. Due to different designs, in the strict harmonisation as used, several important characteristics had to be excluded from the analysis (e.g., BPD and maternal diabetes). Moreover, the cohorts did not provide detailed information about the administration protocol of some care practices (e.g., for starting and ending CPAP-therapy, nutrition of the infants or growth data at discharge). Some data not collected or lost during the harmonisation (e.g. sociodemographic status, maternal illness) could have influenced the results of this study. Missing values could have also influenced the results, although most of the variables included in this study comprised rates of missing data around 10–15% or below.On one hand, this comparison of both cohorts does not provide a whole picture of changes of the medical and social characteristics of the VPs over time. On the other hand, we analysed data on the basis of identical characteristic definitions between two studies with different study design. This could be a direction for future investigations, both for within-country and cross-country comparisons of characteristics, short-term or long-term morbidities of VPs. Our harmonised dataset from POPS and LOLLIPOP is a strong basis for future comparative analysis with regard to long-term health and development of the selected children.

## Conclusions

This comparative study of two VP-born infant cohorts from 1983 and from 2003 in the same country has identified substantial changes in terms of the rates of IVH, sepsis, apnoea, the use of CPAP, the use of caffeine therapy, and the length of NICU and hospital stay. Studies investigating changes in neonatal morbidity with changes in neonatal care need to consider the sociodemographic changes of the populations over time. Although, we could not identify the effect of sociodemographic parameters on changes in neonatal morbidity and care practices, we cannot state that other potential sociodemographic parameters did not influence the improvements. We found that altered therapeutic approaches may have led to fewer cases of severe IVH and sepsis. Moreover, the improved neonatal care practices (including the use of antenatal corticosteroids, the use of surfactant therapy, and the improved respiratory support) may have contributed to the cost-reducing shortening of the length of NICU (− 50%) and total hospital (− 15%) stays.

Most remarkable is the fact that, despite a considerable increase in the survival rates from 1983 to 2003, the neonatal morbidities of the surviving infants investigated here have not increased. This is promising and may be due to changes implemented in the applied national protocols over the period of time studied.

### Electronic supplementary material

Below is the link to the electronic supplementary material.

Supplementary Material 1

## Data Availability

Data requests can be submitted to the cohort coordinators (POPS: SP; LOLLIPOP: AFB) who will evaluate this request. Metadata of the POPS cohort data are also available on the RECAP data platform (https://recap-preterm.inesctec.pt/cat). SPSS and STATA syntax requests for the current analysis can be submitted to reka.sexty@uni-graz.at.

## Abbreviations

BPD: Bronchopulmonary dysplasia
BW: Birth weight
CI: Confidence interval
CPAP: Continuous positive airway pressure
GA: Gestational age
IVH: Intraventricular haemorrhage
LOLLIPOP: Longitudinal Preterm Outcome Project
NEC: Necrotising enterocolitis
NICU: Neonatal intensive care unit
OR: Odds ratio
PCHC: Preventive child healthcare centre
POPS: Project on Preterm and Small-for-gestational age infants
PPROM: Preterm premature rupture of membranes
ROP: Retinopathy of prematurity
SGA: Small-for-gestational age
VP: Very preterm
